# Ni(II), Cu(II), Mn(II), and Fe(II) Metal Complexes Containing 1,3-Bis(diphenylphosphino)propane and Pyridine Derivative: Synthesis, Characterization, and Antimicrobial Activity

**DOI:** 10.1155/2021/4981367

**Published:** 2021-07-12

**Authors:** Maged S. Al-Fakeh, Gadah A. Allazzam, Naeema H. Yarkandi

**Affiliations:** ^1^Chemistry Department, College of Science, Qassim University, Buraidah 51452, Saudi Arabia; ^2^Taiz University, Taiz, Yemen; ^3^Chemistry Department, Faculty of Applied Science, Umm Al-Qura University, Makkah, Saudi Arabia

## Abstract

Four of the coordination compounds of the general formula, [M(DPPP)(APY)(H_2_O) Cl_2_].xH_2_O, where *M* = Ni(II), Cu(II), Mn(II), and Fe(II) and *x* = 0, 1, or 2 molecules of H_2_O, DPPP = 1,3-bis(diphenylphosphino)propane, and APY = 2-aminopyridine, have been prepared and characterized. The structure of the complexes has been confirmed by elemental analysis, FT-IR, and UV-Vis spectral data. Thermal analysis (thermogravimetry, derivative thermogravimetry, and differential thermal studies) has been used to study the thermal decomposition stages. Biological activity of all synthesized complexes was tested against five bacterial strains and three fungal strains. Bacteria and fungi strains are common contaminants of the environment in Saudi Arabia, some of which are frequently reported from contaminated water, soil, and food.

## 1. Introduction

**Figure 1:**
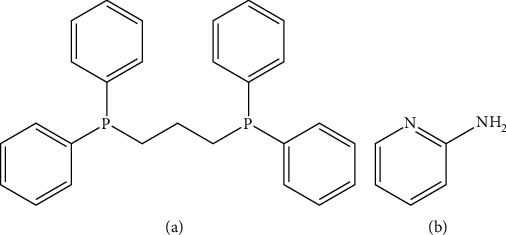
Structure of the ligands. (a) 1,3-Bis(diphenylphosphino)propane. (b) 2-Aminopyridine.

Coordination compounds have been known since the beginning of modern chemistry; also, coordination compounds are so common that their structures and reactions are described as in many ways. The atom within a ligand is bonded to the central metal atom. Especially in hydrometallurgy, the coordination chemistry of the metals participate plays a large role in their solubility and reactivity, as the ore is refined into a precious transition elements. Diphosphine ligands, such as 1,3-bis(diphenylphosphino)propane, are organophosphorus compounds, and these compounds are white solids that are soluble in organic solvents. It is also degraded in air to phosphine oxide and slightly air-sensitive. It is classified as a diphosphine ligand in coordination chemistry and homogeneous catalysis [1–3]. The mixed use of metal complexes containing diphosphines and other ligands, which consist of N or S atoms have been intensively inspected during the past years because of their possibility applications in the fields of light emitting devices, biological activity, and catalysis [4–8]. Additionally, a pronounced attention has been induced by the investigation of the new bioactivity of metal-organic framework materials (BIOMOFs) contained in construction materials as potential antifungal and antibacterial materials (e.g., antifungal glass, antibacterial coating, or antibacterial) [9–14]. Diphosphines are essential ligand backbones, which are coordinated to the metal via monodentate or bidentate manner, and these ligands were used in the development of biologically active metal coordination complexes [15–18]. Pyridine derivatives avail as useful chelating ligands in a large form of inorganic and organometallic applications, and additions act as monodentate ligands that coordinate transition metal ions (N–Ni, N–Cu, Mn–N, and Fe–N) occupy an important position in organometallic chemistry. In addition, there are several reports on pyridine derivative compounds in which the amino group (NH_2_) also participates in coordination [19–23]. In view of the above importance of these ligands and its complexes, we report in this work on the synthesis and characterization of Ni(II), Cu(II), Mn(II), and Fe(II) coordination compounds with DPPP and APY. The structures of the ligands are presented in Figure 1.

## 2. Experimental

### 2.1. Materials

High purity 1,3-bis(diphenylphosphino)propane and 2-aminopyridine were supplied from Sigma Aldrich grade. They were purchased and used without purification.

### 2.2. Physical Measurements

The chemical analyses, CHN, were performed using, Analyischer Functions test Var. El Fab Nr. (11982027) elemental analyzer. FT-IR were recorded as potassium bromide disks (400 to 4000 cm^−1^) with a FT-IR spectrophotometer and the UV-Vis spectra were obtained using a Shimadzu (UV-2101) PC spectrophotometer. Magnetic susceptibility measurements were done on a magnetic susceptibility balance of the type (MSB-Auto). The conductance of the complexes was measured using a conductivity meter model (4310, JENWAY). Thermal analysis of the compounds was carried out in dynamic air on a Shimadzu (DTG 60-H) thermal analyzer at a heating rate (10°C min^−1^).

### 2.3. Antimicrobial Activity of the Complexes

#### 2.3.1. Bacterial and Fungal Strains

The antimicrobial activity of different four complexes extracts was evaluated using five bacterial strains and three fungal strains. Three strains of gram-positive cocci *(Staphylococcus epidermidis, Enterococcus faecalis,* and *Staphylococcus aureus*), two strains of gram-negative bacilli (*Escherichia coli,* and *Pseudomonas aeruginosa*), one stain of yeast-like fungi (*Candida albicans*), and 2 molds (*Aspergillus fumigatus* and *Aspergillus flavus*) were used in this study.

#### 2.3.2. Inoculum Preparation

Bacterial and yeast-like fungal inoculum were prepared from fresh pure cultures in Muller Hinton broth. Each bacterial and yeast-like fungal suspension were compared with 0.5 McFarland standard. Mold samples were inoculated directly into Sabouraud dextrose agar.

#### 2.3.3. Agar Diffusion Assay

The antimicrobial activity of different chemical complex extracts against the selected microorganisms was evaluated using the agar diffusion assay. Each bacterial inoculum was spread on to two Mueller–Hinton agar plates using a sterile cotton swab by lawn culture technique, and the mold samples were inoculated directly onto Sabouraud dextrose agar. After inoculation, wells were made with the help of a sterile cork borer; three wells were made in one of the agar plates for extract numbers 1–3, and four wells were made in the second plate for extract numbers 4–7. Then, each extract (100 *µ*l) was added to already marked well. The plates were then incubated for 18–24 hrs at 37°C. After incubation, we observed the zone of inhibition around the wells and measured the zone diameter in millimeters (mm) by a ruler.

### 2.4. Preparation of the Four Complexes

#### 2.4.1. Preparation of [Ni(DPPP)(APY)(H_2_O)Cl_2_].H_2_O Complex (1)

An aqueous solution (10 mL) of NiCl_2_.6H_2_O (0.46 g, 1.9 mmol) was added to 1,3-bis(diphenylphosphino)propane (DPPP) (0.8 g, 1.9 mmol) of CH_2_Cl_2_ in 10 mL in the presence of NaOH (0.1 M). To the whole solution, an ethanolic solution (10 mL) of 2-aminopyridine (APY) (0.18 g, 1.9 mmol) was added. Pale brown precipitate was separated after refluxing for 3 h. The precipitate was filtered out after being cooled, washed with distilled water and ethanol, and then dried over P_2_O_5_ in a dissector.

#### 2.4.2. Preparation of [Cu(DPPP)(APY)(H_2_O)Cl_2_] Complex (2)

The complex was synthesized by adding the CuCl_2_.2H_2_O solution (0.82 g, 4.8 mmol) in 20 mL distilled water to 2g (4.8 mmol) of 1,3-bis(diphenylphosphino)propane in 10 mL of CH_2_Cl_2_ in the presence of NaOH (0.1 M). Then, to the mixture solution, a 15 mL ethanolic solution of APY (0.45 g, 4.8 mmol) was added immediately. The mixture was refluxed for 3 h and then left aside at room temperature. A light green color was produced and the latter was separated and washed with H_2_O and ethanol.

#### 2.4.3. Preparation of [Mn(DPPP)(APY)(H_2_O)Cl_2_] Complex (3)

The Mn(II) complex was prepared by adding the metal salt MnCl_2_.4H_2_O (0.87 g, 4.4 mmol) dissolved in 20 mL of distilled water with DPPP ligand (1.8 g, 4.4 mmol) dissolved in about 15 mL of a solution (1 : 2 MeOH/CH_2_Cl_2_) in the presence of NaOH. The subsequent process was the addition of a 10 ml ethanolic solution of APY ligand (0.41 g, 4.4 mmol). The mixture was refluxed for about 4 h and then cooled. The dark-brown color formed separated, which was filtered and washed with EtOH and dried over P_2_O_5_.

#### 2.4.4. Preparation of [Fe(DPPP)(APY)(H_2_O)Cl_2_].2H_2_O Complex (4)

A 1,3-bis(diphenylphosphino)propane (2g, 4.8 mmol) was dissolved in 15 mL of a solution (1 : 2 MeOH/CH_2_Cl_2_) in the presence of sodium hydroxide and then the metal salt FeCl_2_ (0.61 g, 4.8 mmol) was added in 15 mL of distilled water followed by addition 15 mL ethanolic solution of 2-aminopyridine (0.45 g, 4.8 mmol), and the resultant product was refluxed for 4 h and then cooled and filtered. Light-yellow precipitate formed and was collected.

## 3. Results and Discussion

The nickel(II), copper(II), manganese(II), and iron(II) coordination compounds were prepared by the reaction of 1,3-bis(diphenylphosphino)propane with metal salts and 2-aminopyridine. The prepared four compounds were found to react in the molar ratio 1 : 1: 1 metal: DPPP: APY. In addition, these compounds are air stable and insoluble in common organic solvents but sparingly soluble in dimethylsulphoxide. The conductivities of the compounds were measured in DMSO using 10^−3^ M solutions of the complexes. The molar conductivity values of the transition metal compounds were 59, 40, 32, and 52 Λm Scm^2^ mol^−1^), respectively. The compositions of the complexes supported by the elemental analysis are recorded together with color in Table 1.

### 3.1. FT-IR Spectra

**Figure 2:**
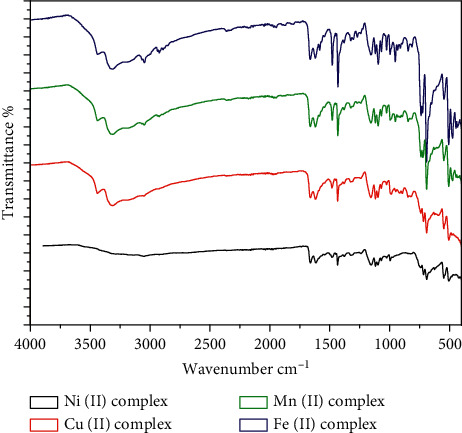
FT-IR spectra of Ni(II), Cu(II), Mn(II), and Fe(II) complexes.

The main infrared spectra of these compounds are listed in Table 2. A comparison of the FT-IR spectra of the complexes 1, 2, 3, and 4 with those of the free DPPP and APY ligands reveals interesting features relating to the metal-ligand (M-L) interactions. From the FT-IR spectra, it is found that the P-PH band, which shows at 1440 cm^−1^ in the spectrum of DPPP, is shifted to a lower wave number (1430–1434 cm^−1^), indicating a sharing of this group in the bonding with the metal ions [24]. The stretching frequencies of *ν*(NH_2_) are observed at 3440 cm^−1^ in all complexes; the spectrum almost undergoes no shift, indicating the nonparticipation of this group in the coordination [25]. On the other hand, the stretching vibration of pyridine group located at 1617 (*ν*) *C* = *C* and 1473 (*ν*) *C* = *N* cm^−1^ in the APY ligand exhibits a notable shift to a wave number (1608–1624) and (1478–1482 cm^−1^) in all complexes, respectively [25]. The C-N stretching in the ring bands of the APY ligand are shifted to lower wave numbers in the range of 1024–1030 cm^−1^ [25]. In the IR spectrum of the free APY, *υ*(C-NH_2_) occurs at 1140 cm^−1^ with no shift in the spectra of the complexes [26]. These results confirm that the pyridine groups are coordinated to the metal ions as a monodentate through the N-atom. The very strong band for the P-C vibration of free forms of phosphine ligand displays a shift from 690 cm^−1^ to a lower wave number (681–694 cm^−1^) [27]. The bands between 2850–2923 and 3020–3046 cm^−1^ are due to the absorption of the phenyl and CH_2_ groups [27]. From these discussions, it is concluded that DPPP coordinates to the metal ions in a bidentate mode through phosphorus atoms. A broad diffused band with medium intensity located in the range 3488–3496 cm^−1^ may be assigned to *ν*(OH) in the lattice H_2_O in (1) and (4) complexes [28]. For the complexes 1–4, the *ν*OH stretching vibration of coordinated H_2_O appears at the 3315–3360 cm^−1^ region [29]. The IR spectra of the complexes 1–4 appear as a band at 734–739 cm^−1^ assigned to *ρ*(H_2_O), which indicates the presence of coordinated water [18]. Metal-oxygen, M-nitrogen, and M-phosphorus vibration bands appear at 504–510, 470–482, and 432–448 cm^−1^ district, respectively (Figure 2) [30, 31].

### 3.2. Electronic Spectra

The UV-Visible spectra of the complexes have been registered in dimethylsulphoxide. The results are manifested in Table 3. The spectra show two distinct bands in the ranges 36,764–39,840 cm^−1^ and 29,325–32,573 cm^−1^, which attributed to *π* ⟶ *π*∗ and *n* ⟶ *π*∗ transitions within DPPP and APY moieties, respectively [27, 32, 33]. On the other hand, in the visible region of spectra, there are characteristic bands attributed to d-d transitions for Ni(II), Cu(II), Mn(II), and Fe(II). For Ni(II), the band is located at the 19,920 cm^−1^ region which is assigned to d-d transition. This band is typical for the octahedral Ni(II) complexes. In the visible spectra of Cu(II), Mn(II), and Fe(II) complexes, the d-d bands are observed in 20,161, 20,040, and 19,841 cm^−1^, respectively, as expected for octahedral Cu(II), Mn(II), and Fe(II).

### 3.3. Magnetic Moments

**Figure 3:**
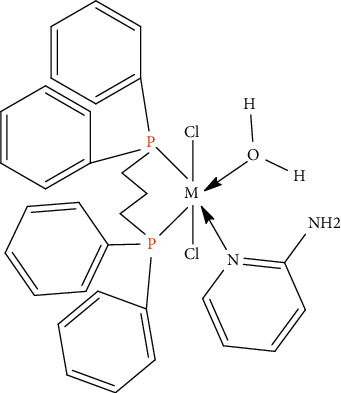
Structure of complexes [M(DPPP)(APY)(H_2_O)Cl_2_].*x*H_2_O, where *M* = Ni(II), Cu(II), Mn(II) or Fe(II) and *x* = 0, 1, or 2 molecules of H_2_O.

**Figure 4:**
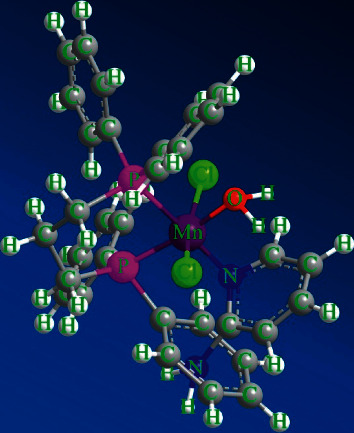
A perspective view of the complete coordination around Mn(II) complex.

The magnetic moments of the complexes were measured. The Ni(II) complex has a magnetic moment located in the range 3.20 BM. As foretelled for a high spin d^8^ system with two unpaired electrons which falls in the range expected for octahedral Ni(II) compounds [34]. Copper(II) complex gave a value of magnetic moment 1.74 BM [34]. The magnetic moment of the iron complex (5.46 BM) measured at room temperature suggests a high spin d^6^ configuration of octahedrally coordinated Fe^2+^ ions [35]. In the case of Mn(II) a compound the value of the magnetic moment 5.30 BM typical for high spin d^5^ system with five unpaired electrons with octahedral arrangement around Mn(II) [34, 36]. From the foregoing data, the structure of the compounds can be postulated as follows (Figures 3 and 4).

### 3.4. Thermal Analysis Studies

**Figure 5:**
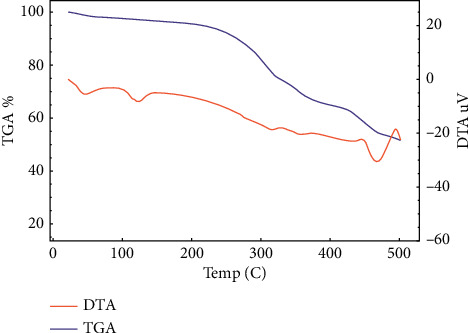
TGA and DTA thermograms of Ni(II) complex.

**Scheme 1:**
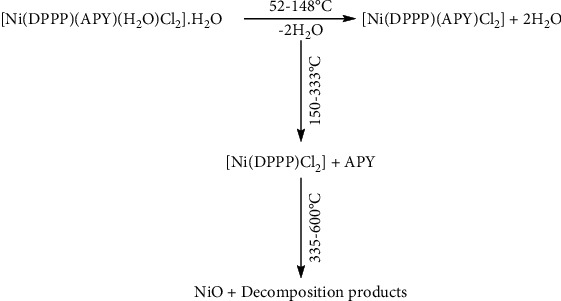
The decomposition stages for nickel(II) complex in dynamic air.

Regarding [Ni(DPPP)(APY)(H_2_O)Cl_2_].H_2_O complex, its decomposition proceeds in five steps (Figure 5). The first and second correspond to the loss of one crystalline H_2_O and one coordinated H_2_O molecule (calc. 5.36%, found 4.92%) (DTG peak at 72, 122°C), for which a broad, endothermic peak appears in the DTA curve at 74 and 124°C. The third and fourth mass loss is concerned to the expulsion of the 2-aminopyridine ligand and chlorine (calc. 24.54%, found 23.69%) (DTG peak at 312, 373°C). This step is marked on the DTA curve by an exothermic effect at 314 and 375°C. The subsequent steps are the decomposition products of the remainder and the rest of the organic ligand. The residue is assigned to be NiO as indicated by the mass loss (calc. 11.10%, found 10.24%) (Scheme 1).

Regarding [Cu(DPPP)(APY)(H_2_O)Cl_2_] complex, the TG, DTG, and DTA curves of this compound show that the thermal decomposition processes involve four stages. The first stage occurs in the temperature range 58–145°C due to the release of the H_2_O molecules (calc. 2.73%, found 2.52%). At this step, a DTG peak appears at 70°C and a broad endothermic effect is recorded in the DTA trace at 73°C. The second step represents a detachment of APY ligand as indicated by mass loss consideration (calc. 14.27%, found 14.05%). This step is manifested in the DTG curve as a peak at 258°C and the DTA trace furnishes an exothermic effect at 260°C. The decomposition of the rest complex proceeds in the next step leaving a stable residue of CuO (calc. 12.06%, found 11.56%).

Regarding [Mn(DPPP)(APY)(H_2_O)Cl_2_] complex, the compound undergoes a stepwise decomposition in three distinct steps of mass loss, namely, at 54–106, 107–320, and 322–600°C. The first step is consistent with the release of H_2_O molecules (calc. 2.77%, found 2.45%). It has a DTG peak at 80°C corresponding to an endothermic peak at 82°C in the DTA trace. In the 2nd step, elimination of the (APY) ligand occurs (calc. 14.46%, found 13.98%). A DTG peak at 296°C with a corresponding broad exothermic peak at 298°C in the DTA trace is observed. The third step represents the thermal decomposition of the rest of the complex. The final product of the compound is compatible with MnO (calc. 10.90%, found 9.97%).

Regarding [Fe(DPPP)(APY)(H_2_O)Cl_2_].2H_2_O complex, the thermogram of [Fe(DPPP)(APY)(H_2_O)Cl_2_].2H_2_O consists of four decomposition stages, namely, at 57–165, 167–278, 280–402, and 404–600°C. The 1st mass loss correlates well with the corresponding of three water molecules, namely, two crystalline and one coordinated, and this may be attributed to an ion-dipole interaction between iron and water (calc. 7.86%, found 7.27%). This step (DTG peak at 81°C) is characterized by an endothermic peak in the DTA curve at 83°C. The second, third, and fourth steps correspond to the decomposition products of the remaining ligands. The final product is assigned to be FeO as indicated by the mass loss in the TG curve (calc. 10.44%, found 10.32%).

### 3.5. Kinetic Studies

**Figure 6:**
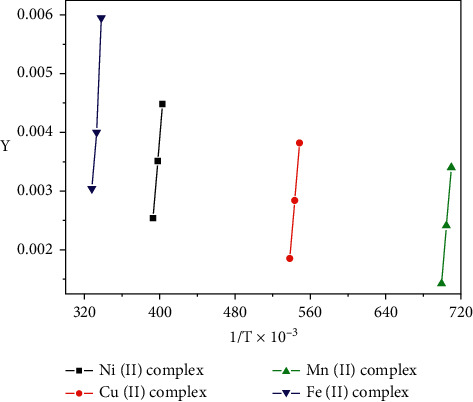
Coats–Redfern plot for the complex 2^nd^ step in dynamic air.

**Figure 7:**
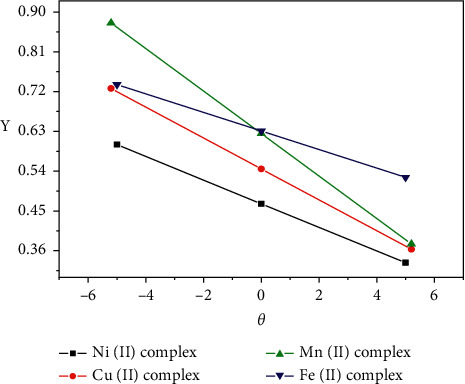
Horowitz–Metzger plot for complex 2^nd^ step in dynamic air.

**Figure 8:**
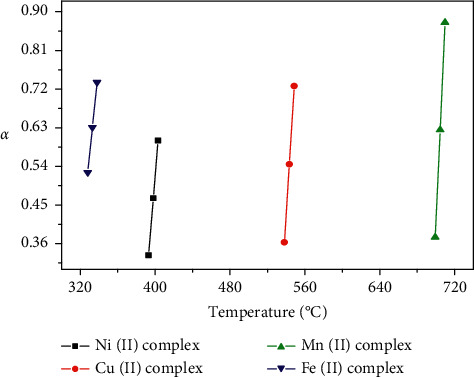
Fraction decomposed (*α*) and temperature plots (*T*) of the complexes.

Nonisothermal kinetic study of the four coordination compounds was carried out applying the Coats–Redfern and Horowitz–Metzger methods (Figures 6–8). The kinetic parameters for all complexes are calculated for the first, second, and third steps according to the Coats–Redfern method and are cited in Tables 4 and 5.

### 3.6. Decomposition Rate and Stability of the Complexes

The decomposition rates of the four complexes have concluded the plotting of the fraction decomposition (*α*) against the temperature (*T*) of the decomposition for the first stage as shown in (Figure 8)

The stabilities of the Ni(II), Cu(II), Mn(II), and Fe(II) coordination complexes could be correlated. In addition to that, the following are the stability orders of the four complexes and the decomposition rates are based on the following:

- The stability order at the initial temperature of the 1^st^ stage of the anhydrous complexes
- The temperature of the inflection point
- DTG maximum temperatures (decomposition rate) and initial temperatures (sequence of stability) that are in parentheses
- The regression of the curves (*α* against *T*) indicates that the complexes decompose by various decomposition rates based on the respective metal ions

(−Ve) ∆*S*^∗^ values for the different steps of decomposition of nickel(II), copper(II), manganese(II), and iron(II) compounds propose that the activated complexes are in higher order than the reactants and also that the reactions are slower than the normal level. The different numbers of enthalpies (∆*H*^∗^) and free energy (∆*G*^∗^) of the four compounds turn on the effect of the structure, each of the metal ions on the thermal stability of the coordination compounds. The (*a* +Ve) values of ∆*G*^∗^ indicated that during the decomposition, reaction is not spontaneous (Table 6).

### 3.7. X-Ray Powder Diffraction of the Four Complexes

**Figure 9:**
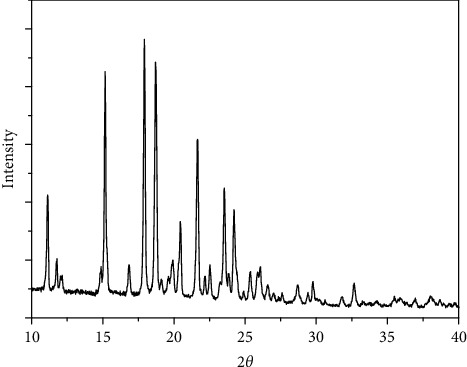
XRD of nickel(II) complex.

**Figure 10:**
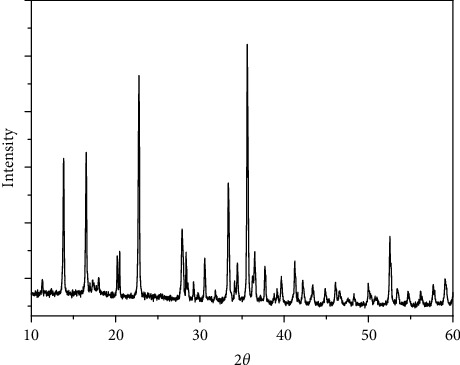
XRD of copper(II) complex.

**Figure 11:**
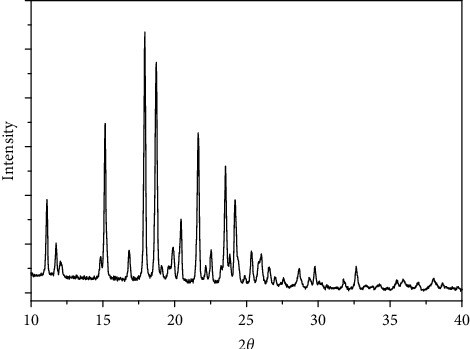
XRD of manganese(II) complex.

**Figure 12:**
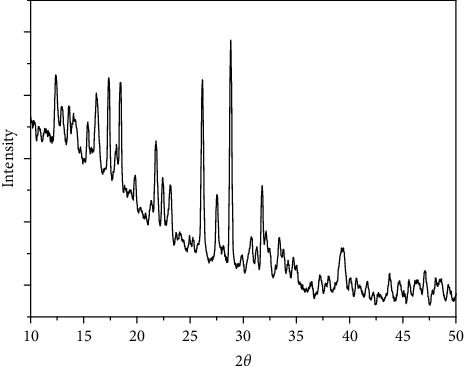
XRD of iron(II) complex.

The X-ray powder diffraction types were recorded for the complexes 1–4. The diffraction shapes indicate that the four compounds are crystalline (Figures 9–12). The crystal data for nickel(II), copper(II), Mn(II), and Fe(II) mixed-ligand coordination complexes belong to the monoclinic, triclinic, and monoclinic crystal system, analyzed by Scherrer's equation. The crystal data for all compounds are listed in Table 7.

### 3.8. Biological Activity of the Coordination Compounds

**Figure 13:**
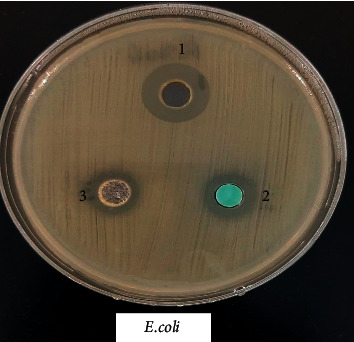
Microbiological screening of Ni(II), Cu(II) and Mn(II) complexes against *E.coli.*

**Figure 14:**
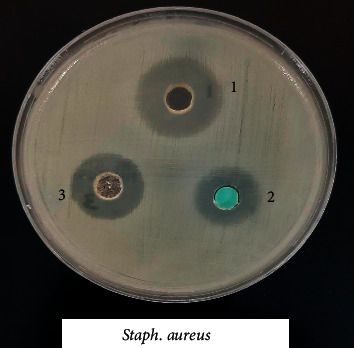
Microbiological screening of Ni(II), Cu(II) and Mn(II) complexes with *S. aureus.*

**Figure 15:**
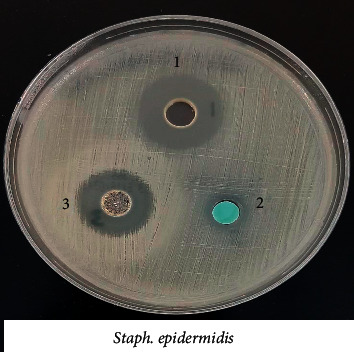
Microbiological screening of Ni(II), Cu(II) and Mn(II) complexes with *S. epidermidis.*

**Figure 16:**
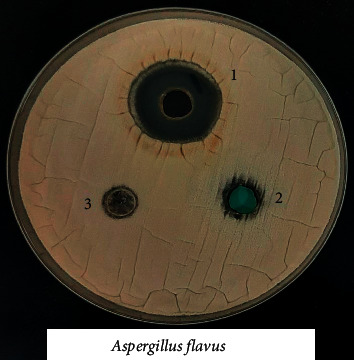
Microbiological screening of Ni(II), Cu(II) and Mn(II) complexes against *Aspergillus flavus.*

**Figure 17:**
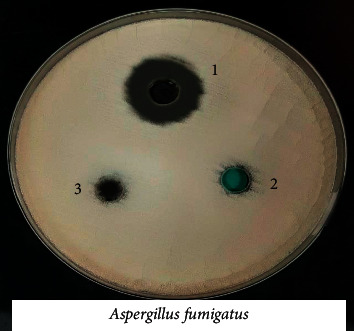
Microbiological screening of Ni(II), Cu(II) and Mn(II) complexes against *Aspergillus fumigatus.*

**Figure 18:**
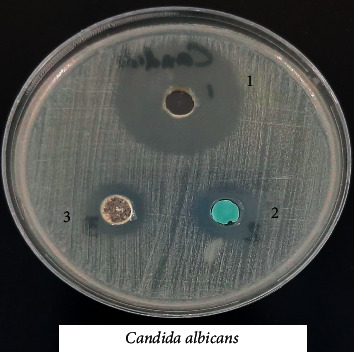
Microbiological screening of Ni(II), Cu(II) and Mn(II) complexes against *Candida albicans.*

Prepared compounds of nickel(II), copper(II), manganese(II), and iron(II) were tested for *in vitro* antimicrobial activity and evaluated against selected bacterial and fungal strains (Figures 13–18). Three strains of gram-positive cocci (*Staphylococcus epidermidis, Enterococcus faecalis,* and *Staphylococcus aureus*), two strains of gram-negative Bacilli (*Pseudomonas aeruginosa* and *Escherichia coli*), one stain of yeast like fungi (*Candida albicans*), and 2 molds (*Aspergillus fumigatus* and *Aspergillus flavus*) were used. The data showed that all complexes have good activity against bacterial strains. Yeast-like fungal stain and mold strains showed good activity mainly toward Ni(II), Cd(II), and Co(II) complexes. Gram-positive bacteria were found to be more sensitive to all tested extracts with significantly higher zones of inhibition than those of gram-negative bacteria and fungal strains. Nickel(II), cadmium(II), and cobalt(II) complexes show higher inhibition zone than the other tested extract complexes as stated in Table 8. The zone of inhibition around the wells is measured in millimeters (mm) by a ruler.

## 4. Conclusion

A number of new one-dimensional nickel(II), copper(II), manganese(II), and iron(II) metal supramolecular coordination compounds of 1,3-bis(diphenylphosphino)propane and 2-aminopyridine have been prepared and characterized by various spectral and physical techniques. From the X-ray analysis, different crystal systems, monoclinic, triclinic, and orthorhombic system for the complexes, were found. The results of antimicrobial activity observed good biological activity for the four compounds, but the nickel(II) complex was over the other three complexes.

## Figures and Tables

**Table 1:** Color, elemental analysis, and decomposition point of the mixed ligand complexes.

Complex	MF MW	Color	Found (Calcd.%)		
			C	H	N
[Ni(DPPP)(APY)(H_2_O)Cl_2_].H_2_O 1	C_32_H_36_N_2_NiP_2_O_2_Cl_2_ 672.2	Pale brown	58.42 (57.17)	6.02 (5.39)	4.36 (4.16)
[Cu(DPPP)(APY)(H_2_O)Cl_2_] 2	C_32_H_34_N_2_CuP_2_OCl_2_ 659.02	Light green	58.68 (58.31)	5.72 (5.19)	4.52 (4.24)
[Mn(DPPP)(APY)(H_2_O)Cl_2_] 3	C_32_H_34_N_2_MnP_2_OCl_2_ 650.41	Dark brown	60.65 (59.08)	5.84 (5.26)	4.78 (4.30)
[Fe(DPPP)(APY)(H_2_O)Cl_2_].2H_2_O 4	C_32_H_38_N_2_FeP_2_O_3_Cl_2_ 687.38	Light yellow	56.28 (55.91)	6.05 (5.57)	4.35 (4.07)

**Table 2:** The infrared spectral data (cm^−1^) of the ligand and their complexes.

Assignment	DPPP	APY	Ni(II) complex	Cu(II) complex	Mn(II) complex	Fe(II) complex
*υ*O-H_lattice water_	—	—	3488-	—	—	3496-
*υ*O-H_coordinated water_			3320	3315	3338	3360
*υ*C=C	—	1617	1615	1624	1622	1608
*υ*C=N	—	1473	1479	1482	1478	1480
P-PH	1440	—	1434	1433	1430	1430
*υ*C-N_in ring_	—	1028	1026	1030	1024	1027
*ρ*OH_2_	—	—	738	739	734	736
P-C	690	—	694	691	687	681
M-O	—	—	510	504	505	507
M-N	—	—	482	470	474	476
M-P	—	—	448	440	432	434

*υ*: stretching and *ρ*: rocking.

**Table 3:** Electronic spectral data of free ligand and its complexes.

Ligands and complexes	*ν*_max_ (cm^−1^)	Assignment	*µ*_eff_. (BM)
DPPP	40,000	*π* ⟶ *π*^∗^	—
APY	31,746	*n* ⟶ *π*^∗^	—
Ni(II) complex	19,920	d-d transition	3.20
	31,948	*n* ⟶ *π*^∗^	
	36,764	*π* ⟶ *π*^∗^	
Cu(II) complex	20,161	d-d transition	1.74
	29,325	*n* ⟶ *π*^∗^	
	39,840	*π* ⟶ *π*^∗^	
Mn(II) complex	20,040	d-d transition	5.30
	32,573	*n* ⟶ *π*^∗^	
	36,900	*π* ⟶ *π*^∗^	
Fe(II) complex	19,841	d-d transition	5.46
	31,847	*n* ⟶ *π*^∗^	
	37,037	*π* ⟶ *π*^∗^	

**Table 4:** Kinetic parameters for the thermal decomposition of Ni(II) and Cu(II) complexes using nonmechanistic equations in dynamic air by Coats–Redfern equation.

Step	[Ni(DPPP)(APY)(H_2_O)Cl_2_].H_2_O				[Cu(DPPP)(APY)(H_2_O)Cl_2_]			
	*n*	*r*	*E* kJ mol^−1^	*Z* s^−1^	*n*	*r*	*E* kJ mol^−1^	*Z* s^−1^
1^st^	0.00	0.9968	36.70	7.24 × 10^2^	0.00	0.9992	39.15	7.76 × 10^2^
	0.33	0.9946	49.63	10.00 × 10^2^	0.33	0.9999	46.71	9.33 × 10^2^
	0.50	**1.0000**	57.62	11.48 × 10^2^	0.50	**1.0000**	51.17	10.23 × 10^2^
	0.66	0.9998	65.80	13.18 × 10^2^	0.66	0.9999	55.46	11.22 × 10^2^
	1.00	0.9981	86.53	17.37 × 10^2^	1.00	0.9993	65.57	13.18 × 10^2^
	2.00	0.9893	169.64	35.48 × 10^2^	2.00	0.9958	102.21	20.89 × 10^2^

2^nd^	0.00	0.9961	74.17	14.79 × 10^2^	0.00	0.9574	17.07	3.46 × 10^2^
	0.33	0.9972	83.68	16.59 × 10^2^	0.33	0.9647	19.99	3.98 × 10^2^
	0.50	0.9980	89.07	17.78 × 10^2^	0.50	0.9673	21.54	4.26 × 10^2^
	0.66	0.9984	94.14	19.05 × 10^2^	0.66	0.9703	23.22	4.57 × 10^2^
	1.00	0.9989	105.55	21.37 × 10^2^	1.00	0.9753	26.91	5.42 × 10^2^
	2.00	**1.0000**	144.22	29.51 × 10^2^	2.00	**0.9859**	40.09	7.94 × 10^2^

3^rd^	0.00	**0.9994**	29.22	5.75 × 10^2^	0.00	0.9978	17.40	3.46 × 10^2^
	0.33	0.9969	34.03	6.76 × 10^2^	0.33	0.9992	21.49	4.26 × 10^2^
	0.50	0.9951	36.76	7.24 × 10^2^	0.50	0.9997	23.86	4.78 × 10^2^
	0.66	0.9929	39.59	7.94 × 10^2^	0.66	0.9999	26.15	5.24 × 10^2^
	1.00	0.9873	46.12	9.12 × 10^2^	1.00	**1.0000**	31.76	6.30 × 10^2^
	2.00	0.9678	70.27	14.12 × 10^2^	2.00	0.9983	52.30	10.47 × 10^2^

Bold values of *r* represent the best fit values of *n*, *E*, and *Z*.

**Table 5:** Kinetic parameters for the thermal decomposition of Mn(II) and Fe(II) complexes using nonmechanistic equations in dynamic air by Coats–Redfern equation.

Step	[Mn(DPPP)(APY)(H_2_O)Cl_2_]				[Fe(DPPP)(APY)(H_2_O)Cl2].2H_2_O			
	*n*	*r*	*E* kJ mol^−1^	*Z* s^−1^	*n*	*r*	*E* kJ mol^−1^	*Z* s^−1^
1^st^	0.00	**0.9951**	14.92	3.01 × 10^2^	0.00	0.9926	25.16	5.01 × 10^2^
	0.33	0.9909	18.16	3.71 × 10^2^	0.33	0.9947	31.48	6.30 × 10^2^
	0.50	0.9886	20.07	3.98 × 10^2^	0.50	0.9961	35.09	6.91 × 10^2^
	0.66	0.9860	21.98	4.36 × 10^2^	0.66	0.9968	38.89	7.76 × 10^2^
	1.00	0.9812	26.57	5.24 × 10^2^	1.00	0.9978	47.34	9.33 × 10^2^
	2.00	0.9664	43.24	8.70 × 10^2^	2.00	**0.9996**	78.89	15.8 × 10^2^

2^nd^	0.00	0.9926	33.75	6.76 × 10^2^	0.00	**0.9934**	128.37	25.70 × 10^2^
	0.33	0.9970	41.08	8.12 × 10^2^	0.33	0.9920	140.46	28.18 × 10^2^
	0.50	0.9984	45.26	8.91 × 10^2^	0.50	0.9917	146.58	29.51 × 10^2^
	0.66	0.9994	49.73	10 × 10^2^	0.66	0.9913	152.69	30.90 × 10^2^
	1.00	**1.0000**	60.19	12.02 × 10^2^	1.00	0.9904	166.68	33.88 × 10^2^
	2.00	0.9952	100.23	19.95 × 10^2^	2.00	0.9870	210.84	41.68 × 10^2^

Bold values of *r* represent the best fit values of *n*, *E*, and *Z*.

**Table 6:** Thermodynamic parameters for the thermal decomposition of the compounds.

Complex	Step	Δ*S*^∗^ kJ mol^−1^K^−1^	Δ*H*^∗^ kJ mol^−1^	Δ*G*^∗^ kJ mol^−1^
Ni(II) complex	1^st^	−190.92	33.98	96.41
		−188.23	46.91	108.46
		−187.09	54.90	116.07
		−185.94	63.08	123.88
		−183.64	83.81	143.86
		−177.70	166.92	225.02

Cu(II) complex	1^st^	−190.10	36.50	96.91
		−188.57	44.06	103.98
		−187.81	48.52	108.20
		−187.04	52.81	112.25
		−185.70	62.92	121.93
		−181.87	99.56	157.35

Mn(II) complex	1^st^	−203.52	9.77	135.66
		−201.78	13.01	137.83
		−201.19	14.92	139.37
		−200.43	16.83	140.81
		−198.91	21.42	144.46
		−194.69	38.09	158.52

Fe(II) complex	1^st^	−194.13	22.39	87.03
		−192.23	28.71	92.72
		−191.46	32.32	96.07
		−190.49	36.12	99.55
		−188.96	44.57	107.49
		−184.56	76.12	137.57

**Table 7:** X-ray powder diffraction crystal data of complexes.

Parameters	Ni(II) complex	Cu(II) complex	Mn(II) complex	Fe(II) complex
Empirical formula	C_32_H_36_N_2_NiP_2_O_2_Cl_2_	C_32_H_34_N_2_CuP_2_OCl_2_	C_32_H_34_N_2_MnP_2_OCl_2_	C_32_H_38_N_2_FeP_2_O_3_Cl_2_
Formula weight	672.2	659.02	650.41	687.38
Crystal system	Triclinic	Monoclinic	Triclinic	Monoclinic
a (Å)	15.509	13.140	15.503	11.491
b (Å)	16.785	9.857	16.788	7.393
c (Å)	10.223	6.018	10.224	9.781
Alfa (°)	103.303	90.00	103.295	90.00
Beta (°)	96.349	103.20	96.342	117.25
gamma (°)	105.685	90.00	105.656	90.00
Volume of unit cell (Å3)	2450.6	758.99	2450.9	738.7

**Table 8:** Microbiological screening of the complexes.

Tested organisms	Ni(II)	Cu(II)	Mn(II)	Fe(II)
*Staphylococcus aureus*	24	17	21	16
*Staphylococcus epidermidis*	23	11	22	15
*Enterococcus faecalis*	24	13	20	15
*Escherichia coli*	18	14	14	13
*Pseudomonas aeruginosa*	16	13	11	13
*Candida albicans*	35	15	15	16
*Aspergillus fumigatus*	19	—	—	—
*Aspergillus flavus*	17	—	—	—

Erythromycin (E15)^*∗*^	24 mm			

Ketoconazole (KET 50)^*∗∗*^	25 mm			

^*∗*^Control of antibacterial; ^*∗∗*^control of antifungus.

## Data Availability

The data used to support the findings of this study are available from the corresponding author upon request.
